# Development of a 2-Nitrobenzoate-Sensing Bioreporter Based on an Inducible Gene Cluster

**DOI:** 10.3389/fmicb.2018.00254

**Published:** 2018-02-14

**Authors:** Satamita Deb, Soumik Basu, Achintya Singha, Tapan K. Dutta

**Affiliations:** ^1^Department of Microbiology, Bose Institute, Kolkata, India; ^2^Department of Physics, Bose Institute, Kolkata, India

**Keywords:** *Cupriavidus* sp., bioreporter, inducible gene cluster, enhanced green fluorescent protein, 2-nitrobenzoate, sensitivity, specificity, immunoblot

## Abstract

Based on the sole information of structural genes of the 2-nitrobenzoate (2NBA) utilizing catabolic gene cluster (*onbX1X2FCAR1EHJIGDBX3*), 2NBA-sensing bioreporters were constructed by incorporating *egfp* into the *onb* gene cluster of *Cupriavidus* sp. strain ST-14. Incorporation of reporter gene in proximal to the hypothesized promoter region in conjunction with the disruption of the gene encoding inducer-metabolizing enzyme was turned out to be advantageous in reporter gene expression at low inducer concentration. The bioreporter strain was capable of expressing EGFP from the very 1st hour of induction and could detect 2NBA at (sub) nanomolar level exhibiting a strict specificity toward 2NBA, displaying no response to EGFP expression from its *meta*- and *para*-isomers as well as from a number of structurally related compounds. The present study is a successful demonstration of the development of a 2NBA-sensing bioreporter with respect to ease of construction, inducer specificity, and sensitivity, without prior knowledge of the associated inducer-responsive promoter-regulator elements. The present approach can be used as a model for the development of bioreporters for other environmental pollutants.

## Introduction

Various anthropogenic impacts based on agricultural and industrial practices, often introduce a variety of organic pollutants including nitroaromatic compounds into the environment at a concentration toxic to existing life forms (Kovacic and Somanathan, 2014). Moreover, upon accumulation over periods, these substances enter into higher eukaryotes including humans via the food chain and contaminated drinking water, thereby disrupting their normal physiological functions (Kelly et al., 2007). Among the wide array of nitroaromatic compounds, nitrobenzoates are widely used in the production of dyes, plastics, explosives, pharmaceuticals, polyurethane foams, elastomers, and pesticides (Peres and Agathos, 2000; Ju and Parales, 2010). These compounds are lethal to living beings because of their genotoxicity, mutagenicity, and hematologic toxicity (Grummt et al., 2006; Arora and Sharma, 2015). Ongoing environmental pollution by these compounds is therefore a worldwide problem and of great interest for remediation.

In spite of being toxic and lethal to higher organisms, bacteria, the most ubiquitous organism in the ecosystem, encounter such pollutants soon after they get released into environment, and often evolve in order to survive, by expanding their capability to utilize these pollutants as carbon sources. Nevertheless, the degradative ability of bacteria is often exploited to clean up pollutants from environment by an eco-friendly and cost-effective process, termed bioremediation (Jørgensen, 2007; Ghosal et al., 2016). Despite the advantages of bioremediation over traditional physicochemical processes (Azubuike et al., 2016), on the whole, it is extremely important to identify and prioritize bioremediation sites based on the quantitative analysis of the load of a particular contaminant and its subsequent levels at different stages of bioremediation.

In the field of analytics, there are a number of precise and sensitive hyphenated chemical techniques but they suffer from various practical concerns such as the need for pre-treatment of samples, use of costly bulky instruments, along with engagement of expert personnel to handle them, and the inability to analyze bioavailable concentrations (Harms et al., 2006; Tecon and Van der Meer, 2008). An alternative is to utilize, reportable biological systems, generally termed ‘biosensors,’ that can be easily handled in a cost-effective manner and can readily sense biologically relevant concentration of pollutants directly, without the need to pre-treat the samples. Biosensors are effective and easy analytics, comprise of a sensory element that specifically interacts with the analyte of interest and passes on the information to a detectable reporting system (Rogers, 2006; Chambers et al., 2008). In spite of the existence of multiple biosensor systems, like *ex vivo* enzymatic assays or antibody affinity assays (Eun-Hyung and Soo-Youn, 2010; Sassolas et al., 2012; Sharma et al., 2016), whole cell bioreporters have gained a great deal of importance due to their ease of use, maintenance and cost-effectiveness (Rogers, 2006; Xu et al., 2013; Hasan et al., 2014). In general the construction of whole cell bioreporters involves the cloning of the promoter-operator-regulator of an inducible catabolic operon into a promoterless reporter plasmid, upstream of reporter gene (that encodes a protein or enzyme that can be easily detected or measured) followed by transformation into a suitable host, mostly *Escherichia coli* (Harms et al., 2006; Tecon and Van der Meer, 2008; Robbens et al., 2010). Alternatively, the reporter gene can be inserted into an inducible catabolic operon of host chromosome/megaplasmid where the expression of reporter gene is under the control of an associated promoter-operator-regulator and inducer molecule (Trögl et al., 2012; Xu et al., 2013; Hong and Park, 2014; Yagur-Kroll et al., 2015). However, unlike the plasmid-based construct in *E. coli* or other heterologous strains, host chromosome-integrated bioreporters ensure a stable construct, that can be easily maintained and circumvents issues associated with analyte transport and compatible sigma factors (Rysz et al., 2013; Muhr et al., 2016).

**FIGURE 1:**
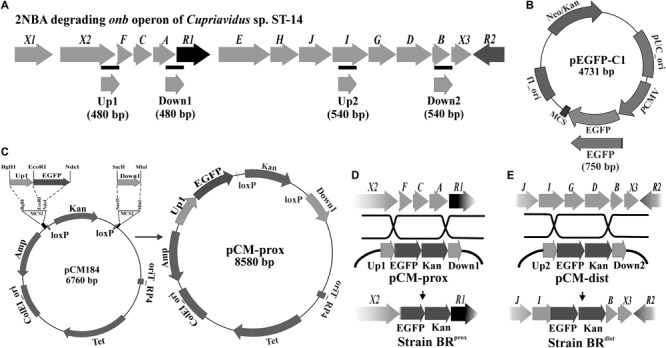
Construction of 2NBA bioreporter strains. **(A)** Genetic organization of the 2NBA degrading *onb* gene cluster in *Cupriavidus* sp. strain ST-14. Black bars indicate the genomic positions of the amplified Up and Down fragments used to construct the recombination plasmids. **(B)** The reporter gene EGFP was PCR amplified using pEGFP-C1 plasmid as template. **(C)** pCM184 was used as the vector backbone in which the Up1, EGFP and Down1 fragments were cloned in the depicted orientation to construct pCM-prox. The pCM-dist plasmid was constructed similarly using Up2, EGFP and Down2 fragments. **(D)** Recombination events occurred between pCM-prox and the ST-14 chromosome with the insertion of EGFP/Kan cassette in the place of *onbFCA* segment to construct the bioreporter strain BR^prox^. **(E)** Recombination between pCM-dist and ST-14 chromosome resulted in the construction of the bioreporter strain BR^dist^ by disrupting the *onbIGDB* segment.

Recently, *Cupriavidus* sp. strain ST-14 capable of degrading 2-nitrobenzoate (2NBA) was characterized for the presence of a 2NBA-inducible *onb* catabolic gene cluster, illustrating biochemical and molecular information on the metabolic pathways of degradation of 2NBA (**Figure 1A**). The core *onb* cluster (*onbFCAR1EHJIGDB*) was found to comprise of 11 unidirectional ORFs that include ten structural genes, *onbA* through *onbJ*, and a putative regulatory protein encoding gene, *onbR1*. In the presence of 2NBA, all the catabolic genes of the cluster were observed to transcribe polycistronically. In this catabolic pathway, 2NBA is first metabolized to 2-hydroxylaminobenzoate (HABA) by OnbA, a nitroreductase. HABA is then transformed by the action of a mutase, OnbB to 3-hydroxyanthranilate (3HAA) which is subsequently ring cleaved by 3-hydroxyanthranilate dioxygenase, OnbC to produce 2-amino-3-carboxymuconic-6-semialdehyde. The semialdehyde is further metabolized by the actions of OnbD (decarboxylase) and OnbE (dehydrogenase), to 2-aminomuconate which is further deaminated by OnbF (deaminase) to release ammonia. Finally, the deaminated product, oxalocrotonate is transformed by sequential actions of OnbGHIJ leading to TCA cycle intermediate acetyl-coA (Basu et al., 2016). In the present study, we report the construction of a highly specific and sensitive 2NBA-sensing bioreporter by strategically integrating a reporter gene into 2NBA-inducible gene cluster of strain ST-14 based on the structural gene information, even without the need for identifying the exact location of the associated promoter-operator sequence or the role of the encoded regulatory protein.

## Materials and Methods

### Chemicals and Reagents

Chemicals of the highest purity were used for the induction assays, and were obtained from Sigma–Aldrich, United States. Reagents used for plasmid isolation, DNA purification, restriction digestion, ligation and reverse transcription were purchased from Thermo Fischer Scientific, United States. Agarose, acrylamide, bis-acrylamide, ammonium persulfate, tetramethylethylenediamine (TEMED), ethidium bromide, DNA loading dye and DNA ladder were procured from SRL, India. Protein ladder was purchased from Genetix, India. Unless stated otherwise, all other chemicals and reagents used in this study were of analytical grade and were used without further purification.

### Strains and Culture Conditions

Wild type and mutant strains of *Cupriavidus* sp. strain ST-14 were grown overnight aerobically at 28°C either in Luria-Bertani (LB, pH 7.0) broth containing (l^-1^) 10 g Bacto-tryptone, 5 g yeast extract, and 10 g NaCl or in a mineral salt medium (MSM, pH 7.0) containing (l^-1^) 3.34 g K_2_HPO_4_, 0.87 g NaH_2_PO_4_, 2.0 g NH_4_Cl, 200 mg MgSO_4_.7H_2_O, 12 mg FeSO_4_.7H_2_O, 3 mg MnSO_4_.H_2_O, 3 mg ZnSO_4_.7H_2_O, and 1 mg CoCl_2_.6H_2_O supplemented with 2 mM or 7 mM sodium succinate (SRL, India) as carbon source. Solid media contained 1.8% (w/v) agar (HiMedia, India). The *E. coli* strain XL1 Blue, used as cloning host, was grown overnight at 37°C in LB broth or on a LB-agar solid medium. Unless mentioned otherwise, clones of *E. coli* as well as ST-14 mutant strains were grown in media supplemented with 50 μg ml^-1^ of kanamycin.

### Construction of Recombination Plasmids and Reporter Strains

Following the methods of Marx and Lidstrom (2002), two different recombinant plasmids pCM-prox and pCM-dist were constructed using pCM184 as the vector backbone. In both, the reporter gene *egfp* was cloned upstream to the intrinsic *kanamycin* resistance gene. The *egfp* gene was amplified using the EGFP forward and EGFP reverse primers from the pEGFP-C1 plasmid (Clontech, United States) and was inserted into *EcoRI* and *NdeI* sites present at vector MCS1. To prepare pCM-prox reporter plasmid, Up1 and Down1 fragments were amplified from the ST-14 genomic DNA, using individual sets of Up1 forward/reverse primers and Down1 forward/reverse primers. Similarly, during construction of the pCM-dist plasmid, Up2 and Down2 fragments were amplified using Up2 forward/reverse and Down2 forward/reverse primers (**Figure 1A**). Amplified Up1 and Up2 fragments were individually introduced into the *BglII* and *EcoRI* sites of the vector MCS1 whereas the Down1 and Down2 fragments were inserted into the *SacII* and *MluI* sites of MCS2 (**Figure 1C**). These Up and Down fragments are the upstream and downstream flanking sequences (**∼**500 bp each) of the targeted *onb* genes to be replaced by the *egfp/kanamycin* cassette. Sequences and chromosomal location of the different Up and Down fragments as well as of all the primer sequences are listed in Supplementary Table S1. Reporter strains BR^prox^ and BR^dist^ were constructed by electroporating the recombinant plasmids pCM-prox and pCM-dist, respectively (Basu et al., 2016) into electrocompetent wild type ST-14 cells prepared following Taghavi et al. (1994). Insertion of the *egfp/kanamycin* cassette from the plasmids into the chromosomal *onb* gene cluster of ST-14 occurred through allelic exchange via double recombination of the Up and Down sites (**Figures 1D,E**). Sequence integrity of the cloned fragments was confirmed by sequence analysis in an ABI PRISM 3500XL sequencer using ABI sequencing reagents (Applied Biosystems, United States).

### Induction Experiments

Induction experiments were carried out with bioreporter cells grown either in Luria-Bertani (LB) broth or in MSM supplemented with 2 mM or 7 mM of succinate. Bioreporter strains were induced in presence of 2NBA either at 0 h of incubation in the growth media (‘-Wash’ condition) or to pre-grown cell suspension in induction medium (‘+Wash’ condition) such as 50 mM potassium phosphate buffer (pH 7.0), carbon-free MSM or MSM supplemented with 1 mM succinate, acetate, citrate, dextrose, pyruvate or protocatechuate (PCA). Unless stated otherwise, for pre-grown cell suspension, non-induced LB broth-grown overnight culture was used with prior washings (thrice) of cells with 50 mM potassium phosphate buffer (pH 7.0). To determine the minimal induction time for EGFP expression, pre-grown cell suspension of BR^prox^ cells were induced in carbon-free MSM and were harvested at every 1 h interval for a period of 24 h. Again, to evaluate the most appropriate growth phase for EGFP expression, carbon-free MSM was used as the induction medium where the washed BR^prox^ cells were introduced from individual LB broth cultures, after attaining different phases of growth. However, to determine bioreporter specificity and sensitivity as well as for experiments of confocal microscopy and flow cytometry, pre-grown cell suspension of BR^prox^ cells induced in MSM + succinate (1 mM) was used. Unless stated otherwise, inducer (2NBA or other structurally related compounds dissolved in methanol) concentration and induction time were maintained at 1 mM and overnight, respectively.

### Preparation of Cell-Free Extract

For preparation of cell-free extract (CFE), 2NBA induced bioreporter cells were harvested, washed thrice with 50 mM potassium phosphate buffer (pH 7.0), resuspended in the same buffer and lysed through French press (One Shot model 182 constant cell disruption system with an 8.0-ml lysis chamber; Constant System Ltd., United Kingdom) at 30,000 lb/in^2^ (207 MPa) for two cycles. Next, centrifugation at 13,000 rpm for 20 min at 4°C, the supernatant was collected as a CFE (cell lysate). Following Bradford method, protein concentrations of the CFE samples were measured spectrophotometrically at 595 nm using 1 × Bradford reagent (Bio-Rad, Germany) and bovine serum albumin (BSA) as the standard (Bradford, 1976).

### Enzyme Assay

The activity of 2NBA reductase (OnbA) was monitored spectrophotometrically (Cary 100 Bio UV-visible spectrophotometer; Varian, Australia) by measuring the 2NBA-dependent decrease in the absorbance at 340 nm due to the oxidation of NADPH (Basu et al., 2016) using 2NBA-induced (1 mM) cell-free extracts of strains BR^prox^ and BR^dist^. In addition, 3HAA dioxygenase (OnbC) activity was estimated in the cell-free extracts of BR^dist^ cells, induced in presence of 8, 10 or 12 mM of 2NBA, by measuring an increase in absorbance at 360 nm due to ring cleavage of 3HAA (Basu et al., 2016).

### Western Blot Analysis

Unless mentioned otherwise, cell-free extracts containing 50 μg protein, were electrophoresed in a 15% SDS–PAGE gel and transferred onto PVDF membranes (GE Healthcare, United Kingdom) using a wet transfer apparatus (Bio-Rad, Germany). EGFP (27 kDa) was probed with primary antibody, anti-GFP (B-2, sc-9996; Santa Cruz Biotechnology, United States) at 1:3000 dilution and was finally detected with HRP-conjugated goat anti-mouse IgG antibody (7076; Cell Signaling Technology, United States) at 1:5000 dilution. Respective protein bands were visualized with enhanced chemiluminescence (ECL) Western blotting substrate (GE Healthcare, United Kingdom), according to the suppliers’ protocol.

### RNA Isolation, cDNA Preparation, and Reverse Transcription

Total RNA was isolated from 2NBA-induced and uninduced cells of wild type ST-14 and BR^prox^ bioreporter strains using TriZol (Invitrogen, United States). Prior to cDNA preparation, genomic DNA contamination was removed by incubating 1 μg of RNA sample with 1 U of RNase free DNase (Thermo Scientific, United States) for 30 min at 37°C followed by inactivation with 50 mM EDTA at 65°C for 10 min. cDNAs for *16S rRNA, egfp, onbC, and onbD-B* mRNAs were prepared according to the manufacturer’s protocol using RevertAid Reverse transcriptase enzyme (Thermo Scientific, United States) using 1 μg of RNA as template and gene-specific RT reverse primers for the abovementioned genes. To avoid RNA degradation through RNase contamination, Ribolock (Thermo scientific, United States) was added to each of the reaction vials. RNA concentration and purity was determined spectrophotometry at 260 nm and by A_260_/A_280_ ratio, respectively. To understand the transcription profiles of the *onbC* and *onbD-B* gene fragments in the wild type bacteria and of the *egfp* gene in the bioreporter strains, RT-PCR analyses were performed using EGFP_RT_F and EGFP_RT_R, onbC_RT_F and onbC_RT_R, onbD-B_RT_F and onbD-B_RT_R primers. *16S rRNA* was amplified using 16S_RT_F and 16S_RT_R primers and was kept as an endogenous loading control for the wild type ST-14 and mutant (bioreporter) strains, BR^prox^ and BR^dist^. Sequences of all the RT primers are given in the Supplementary Table S1.

### qPCR Analysis

Quantitative PCR (qPCR) was done with 96-well qPCR plates by using ABI 7500 Fast detection system (Applied Biosystems, United States). PCR reactions were carried out with a final volume of 20 μl, using 2 μl of *16S rRNA* and *egfp* cDNA samples as template with 1 μM final concentration of each of the forward and reverse RT primers of the said genes and 8 μl of 2 × SYBR green PCR mix (Applied Biosystems, United States). The PCR program included a 10 min denaturation step at 95°C, followed by 40 cycles of 15 s of denaturation at 95°C and 30 s of primer hybridization at 55°C and 30 s of polymerization at 60°C. Relative gene expression levels were calculated using the comparative threshold amplification cycle (*CT*) by 2*^-ΔΔC_T_^*method (Schmittgen and Livak, 2008). For each set of reactions, the *16S rRNA* gene was kept as an endogenous control whereas cDNAs of uninduced cells were taken as reference samples.

### Fluorimetric Analysis

2NBA induced and uninduced BR^prox^ cells of strain ST-14 were thoroughly washed thrice with 50 mM potassium phosphate buffer (pH 7.0) to remove all traces of inducer present in the medium. Washed cells were resuspended in potassium phosphate buffer so as to achieve an OD_600_ of 1.0. Fluorescence intensity of EGFP of each of these samples (200 μl in micro cuvettes) was measured using a LabRAM HR spectrometer (Jobin Yvon, Japan). An air-cooled argon ion (Ar^+^) laser of wavelength 488 nm was used as the excitation source and a Peltier-cooled charge-coupled-device (CCD) was used as the detector. Each spectrum was taken in 20 s integration time in the emission window of 495–600 nm where the laser power was 0.5 mW. A single point data acquisition at 513 nm was performed to quantify EGFP intensities. Measured fluorescence values were corrected by subtracting background fluorescence values obtained using potassium phosphate buffer alone. Following identical protocol, cell-free extracts (200 μl) prepared from the same cellular samples were also analyzed and the obtained fluorescence values were normalized to a protein concentration of 1 mg/ml.

### Confocal Microscopy

2NBA induced and uninduced cells of the BR^prox^ bioreporter strain of ST-14 were harvested and washed thrice with 50 mM potassium phosphate buffer (pH 7.0), and resuspended in the same buffer to obtain an OD_600_ of 1.0 prior to fixation using 4% paraformaldehyde for 1 h at room temperature followed by heating at 70°C for 15 min (Kniggendorf et al., 2011). For chromosomal staining, 100 μl of these cell suspensions were incubated with 5 μl of DAPI (1 mg ml^-1^) solution (Sigma–Aldrich, United States) for 20 min in dark at room temperature. Treated cells were illuminated using laser specific for DAPI (excitation: 356 nm; emission range: 420–475 nm) or GFP (excitation: 488 nm; emission range: 505–530 nm) and visualized under a confocal microscope (Leica, TCS-SP8, Germany) using 63 × oil immersion lens. To avoid cellular stacking, a 10 μl aliquot of the diluted (OD_600_ = 0.3) fixed cellular samples was used for imaging. All sets of imaging are performed with the confocal microscope using identical parameters.

### Flow Cytometry

Prior to flow cytometric analysis, BR^prox^ cells were individually induced with 100 nM, 100 μM or 1 mM of 2NBA in succinate containing MSM and harvested after overnight incubation. All these cells were washed with filtered 1 × PBS (pH 7.0) and resuspended in the same solution so as to achieve an OD_600_ of 0.5. EGFP expression in the induced cell populations were analyzed using a FACS VERSE flow cytometer (BD, United States). Cells were illuminated with a 15 mW argon ion (Ar^+^) laser (488 nm excitation). To measure fluorescence emission, a 525 BP filter was used to restrict emission wavelength from 505 to 545 nm. Analysis was performed with 50,000 particles. Uninduced cells were taken as EGFP negative control and the population was subjected to gating to specify the P1 population with lower EGFP intensities. In case of induced cell populations, rightward cellular protrusions from the gating of P1 population were counted as P2 populations with relatively high EGFP expression. Whole population of the induced cells was overlaid whereas the stacked representation of the P2 population was demonstrated using FACSUIT software.

### Statistical Analysis

Experiments were performed in at least three biological replicates, with technical repeats for each biological replicate. All data were presented as mean values of three measurements from replicated experimental samples with standard errors. For Western blot and confocal microscopy data, one of the three replicated images for each set of experiments was presented. Protein band intensities were presented as Relative Unit of Densitometry (RUD) ± Standard Error (SE).

## Results

### Construction of Bioreporter Strains and Reporter Gene Expression

The 2NBA utilizing *Cupriavidus* sp. strain ST-14 was shown to harbor the *onb* gene cluster responsible for the complete degradation of the nitroaromatic compound. It has already been observed that the genes in the *onb* cluster (*onbX1X2FCAR1EHJIGDBX3*) are organized in the same direction (**Figure 1**), contiguous with respect to the complete set of 2NBA catabolic genes and are polycistronic in nature (Basu et al., 2016). Therefore, it has been hypothesized that the promoter-operator region should reside somewhere upstream to the first structural gene *onbF*. As transcription of the degradative genes is recognized to be 2NBA inducible (Basu et al., 2016), construction of a functional bioreporter was deemed a viable choice without going into the details of molecular characterization of the associated promoter and regulatory elements.

**FIGURE 2:**
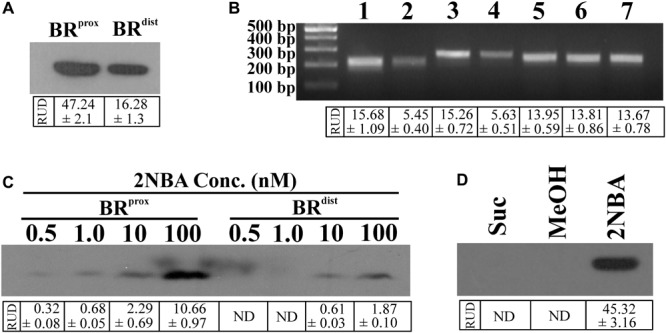
Differential reporter gene expression in constructed bioreporters. **(A)** Western blot analysis to determine the level of EGFP expression in cell lysates (50 μg protein) of the bioreporter strains, BR^prox^ and BR^dist^ induced with 2NBA (1 mM). **(B)** RT-PCR analysis exhibiting the mRNA transcript level of *egfp* gene in 2NBA-induced BR^prox^ (3) and BR^dist^ (4) strains which corroborated the transcriptional profiles of the wild type *onbC* (1) and *onbDB* (2) genes, located at similar positions in the *onb* gene cluster as that of *egfp* gene in BR^prox^ and BR^dist^ strains, respectively. Transcription levels of *16S rRNA* for wild type ST-14 (5) as well as that of BR^prox^ (6) and BR^dist^ (7) mutants were shown as endogenous controls. **(C)** Differential expression of reporter signal in the BR^prox^ and BR^dist^ strains, induced at low concentrations of 2NBA, analyzed by immunoblotting of EGFP using the cell lysates containing 50 μg of protein. **(D)** Western blot analysis to determine EGFP expression in BR^prox^ cells induced in presence of 1 mM 2NBA (dissolved in methanol). Parallel controls depicting EGFP expression in BR^prox^ strain in the absence of 2NBA but in the presence of succinate (Suc) and succinate plus equivalent amount of methanol (MeOH) are shown. In each case, analysis was performed using cell-free extracts containing 50 μg protein. In all these experiments, inducer was added to the growth medium (MSM + 2 mM succinate) at 0 h of incubation (‘–Wash’ condition). Protein and nucleic acid band intensities are presented as RUD (Relative Unit of Densitometry) ± SE (Standard Error) values.

Given the background, the BR^prox^ and BR^dist^mutants of strain ST-14 were constructed by disrupting the chromosomal *onbFCA* and *onbIGDB* gene segments with the incorporation of the fluorescent reporter gene *egfp* by homologous recombination using the protocols as described in the ‘Materials and Methods’ section. Unlike the wild type strain, the identity of these reporter mutants was verified by their kanamycin resistance and presence of the reporter gene using PCR analysis. The two mutants, where the reporter gene is incorporated proximal or distal to the hypothesized promoter region, were tested to observe the effect of induction by 2NBA. When both the strains were induced in presence of 2NBA (1 mM), BR^prox^ mutant produced nearly thrice the amount of EGFP (RUD, 16.28 ± 1.3) compared to that of BR^dist^ (RUD, 47.24 ± 2.1) mutant (**Figure 2A**). This data corroborated the fact that due to polar effects, distal genes are expressed at lower levels (1/3 fold) than that of promoter proximal genes. This was also reflected in the reverse transcription (RT-PCR) analysis depicting the differential levels of the mRNA transcript of *onbC* (proximal gene) compared to that of *onbD*-*onbB* (distal genes) (**Figure 2B**) in the 2NBA induced wild type ST-14 cells. Moreover, the purpose of the construction of the BR^prox^ mutant with impaired *onbA*, the gene encoding the nitroreductase (Basu et al., 2016) responsible for the transformation of 2NBA to 2-hydroxylaminobenzoate (HABA), is to render the mutant incapable of transforming 2NBA. Consequently, at 0.5 and 1.0 nM concentrations of 2NBA, no reporter gene expression was detected in BR^dist^ cells while at 10 and 100 nM concentrations of 2NBA, only 1/4 and 1/6 fold of EGFP expression were revealed, respectively, compared to that in BR^prox^ cells (**Figure 2C**). This can be explained by the fact that in BR^prox^ cells, 2NBA remains unaltered due to OnbA inactivation (Supplementary Figure S1A) and is thus capable of driving prolonged induction in contrast to the BR^dist^ strain where 2NBA is converted to HABA due to the action of functional OnbA (Supplementary Figure S1B). Nevertheless, reporter gene expression was observed solely due to 2NBA-mediated induction and not because of non-specific recognition of succinate (used as carbon source), components of MSM or methanol used as solvent for dissolution of 2NBA (**Figure 2D**). Thus, incorporation of the reporter gene into the promoter-proximal region along with the disruption of the inducer-metabolizing enzyme, is advantageous in reporter gene expression; more specifically, at low inducer concentration. Hence the strain BR^prox^ was selected for further studies.

### Optimization of Media, Growth Phase and Induction Time on Reporter Gene Expression

**FIGURE 3:**
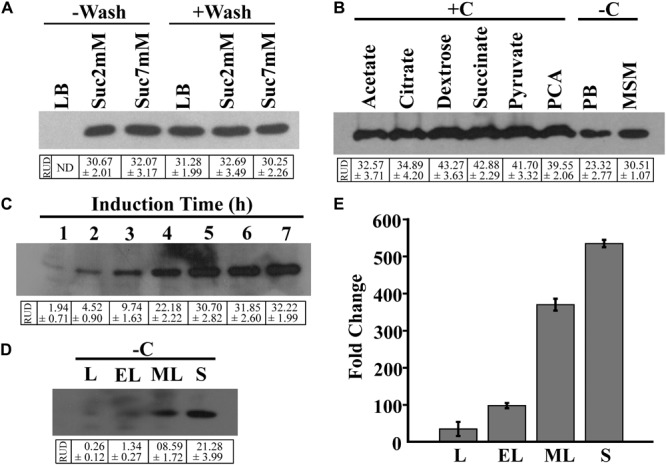
Optimization of reporter expression. **(A)** EGFP expression of 2NBA-induced (1 mM) BR^prox^ cells, grown in LB broth or in MSM supplemented with 2 mM or 7 mM succinate (growth media). Inducer was added either to the growth medium at 0 h of incubation (‘–Wash’ condition) or to the pre-grown cell suspension in carbon-free MSM (induction medium) (‘+Wash’ condition). Pre-grown cell suspension was prepared from overnight grown culture of bioreporter cells in respective medium and subsequent washings of cells with 50 mM phosphate buffer. In each case, 50 μg of protein of respective cell lysates was analyzed by immunoblotting of EGFP. **(B)** EGFP expression profile of pre grown (LB broth-grown) cell suspensions of BR^prox^ strain, induced overnight with 2NBA (1 mM) in 50 mM phosphate buffer (PB, pH 7.0), in carbon-free MSM (–C) or in MSM in presence (+C) of various carbon sources (1 mM). Western blot analysis was performed using 50 μg of protein of respective cell lysates. **(C)** Time-dependent EGFP expression upon 2NBA (1 mM) induction (in carbon-free MSM) of the pre-grown (overnight LB broth-grown) cell suspension, analyzed by Western blotting using cell lysates (100 μg protein) of 2NBA induced cells, harvested at every 1 h interval. **(D,E)** EGFP induction profile of pre-grown (LB broth-grown) cell suspension harvested at lag (L), early-log (EL), mid-log (ML) and stationary (S) phases and induced with 2NBA (1 mM) in carbon-free MSM. Levels of EGFP protein expression **(D)** and *egfp* mRNA transcripts **(E)** in BR^prox^ cells obtained from each of the growth phases, analyzed by Western blot (50 μg protein) and qPCR analysis, respectively. Protein band intensities are presented as RUD (Relative Unit of Densitometry) ± SE (Standard Error) values.

For the growth of the BR^prox^ mutant, LB broth served as the best growth medium in comparison to MSM supplemented with succinate (Supplementary Figure S2). In spite of yielding the highest cell mass, LB broth was not found suitable as an induction medium, failing EGFP expression of cells when induced overnight with 2NBA (‘-Wash’ condition). However, 2NBA-induced overnight culture of BR^prox^ mutant in succinate-containing MSM as growth media (‘-Wash’ condition) exhibited EGFP expression. On the other hand, washed cells of BR^prox^ mutant, pre-grown on LB broth or succinate-containing MSM, upon overnight induction with 2NBA in carbon-free MSM, produced a noticeable amount of EGFP (‘+Wash’ condition) (**Figure 3A**).

**Figure 3B** illustrates 2NBA-mediated induction profiles of pre-grown culture of BR^prox^ mutant in the absence and presence of various carbon sources. 2NBA-induced EGFP expression was noticed both in carbon-containing and in carbon-free induction media. However, under carbon-containing conditions, enhanced levels of EGFP expression were revealed. This may be attributed due to utilization of carbon sources resulting in an enhanced biosynthesis of the reporter protein. Nevertheless, in carbon-containing induction media, the presence of dextrose, pyruvate, succinate or PCA revealed relatively higher levels of EGFP expression than that of other carbon sources used (**Figure 3B**). On the other hand, EGFP expression of pre-grown culture of BR^prox^ mutant, induced in potassium phosphate buffer or carbon-free MSM may be explained due to utilization of cell-accumulated compatible nutrients (Heitzer et al., 1992). It may be mentioned here that the minimum time for EGFP expression was observed to be 1 h based on 2NBA induction of pre-grown culture of BR^prox^ mutant in carbon-free medium. However, the level of EGFP expression increases with time up to 5 h, beyond which expression level gets saturated (**Figure 3C**). 2NBA-mediated induction (in carbon-free MSM for 5 h) profiles of pre-grown BR^prox^ cells for lag, early-log, mid-log and stationary phase culture grown in LB broth are depicted for EGFP expression both at protein as well as mRNA level (**Figures 3D,E**). It is clear that in comparison to lag (RUD, 0.26 ± 0.12), early-log (RUD, 1.34 ± 0.27) and mid-log phase cells (RUD, 8.59 ± 1.72), stationary phase cells (RUD, 21.28 ± 3.99) exhibited an enhanced level of induction.

### Bioreporter Specificity

Unlike 2NBA, induction of the BR^prox^ mutant cells individually in the presence of the 2NBA structural isomers, 3-nitrobenzoate and 4-nitrobenzoate did not exhibit EGFP expression. Similarly, neither of the several other structurally related compounds viz., 5-hydroxy-2-nitrobenzoate, 2,4-dinitrobenzoate, 2-hydroxy-3,5-dinitrobenzoate, benzoate, salicylate (2-hydroxybenzoate), 2,3-dihydroxybenzoate, gentisate (2,5-dihydroxybenzoate), 2-hydroxy-5-methoxybenzoate 2-chlorobenzoate, 2-iodobenzoate, 2-aminophenol, 2-nitrophenol, 2-nitroaniline, 2-nitrotoluene, 2,3-dinitrotoluene, 2,4-dinitrotoluene, 2-nitrobenzyl alcohol, 2-nitrobenzaldehyde, phthalate, 2-nitrobenzoyl chloride, 1-chloro-2-nitrobenzene, 1-fluoro-2-nitrobenzene, 2-nitrophenyl acetate and methyl-2-nitrobenzoate could induce the expression of EGFP (Supplementary Table S2). Inability of all these structurally related compounds to induce the reporter system was confirmed both at the RNA and protein levels, displaying data for 2NBA structural isomers and a few (hydroxy) benzoates (Supplementary Figure S3). These results confirmed that the transcription of the *onb* genes are specifically induced by 2NBA and therefore, the BR^prox^ bioreporter should exclusively monitor 2NBA in the presence of a mixture of structurally related compounds without being impacted by cross-reactivity.

### Bioreporter Sensitivity

**FIGURE 4:**
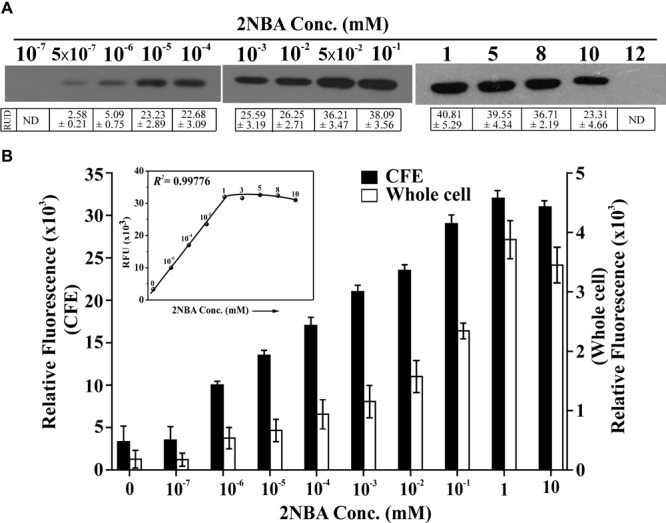
Sensitivity of reporter strain. **(A)** EGFP expression of BR^prox^ mutant following induction with 2NBA in a concentration range of 10^-7^ mM to 12 mM in MSM in presence of 1 mM succinate. In the inducer concentration range of 10^-7^ mM to 10^-1^ mM, 100 μg of protein (cell lysate) was used while in the range of 1–12 mM, 50 μg of protein (cell lysate) was used in Western blot analysis. Protein band intensities are presented as RUD (Relative Unit of Densitometry ± SE (Standard Error) values. **(B)** Relative fluorescence of the 2NBA-induced bioreporter cells as described above was quantified both for whole cell and cell-free extracts (CFE) using fluorescence spectrophotometer. Fluorescence data were given for the emission peak at 513 nm for whole cells or CFE, with 488 nm laser excitation. A concentration vs RFU (Relative Fluorescent Unit) graph for the CFE is given in the inset showing the *R^2^* value for the linear region. Each RFU value (solid circle) is related to the corresponding concentration of 2NBA (mM) shown in the graph. All these experiments were performed with pre-grown (overnight LB-broth grown) cell suspensions induced with 2NBA in MSM + 1 mM succinate (‘+Wash’ condition).

Stationary phase cells of BR^prox^ mutant, induced with 2NBA in the presence of 1 mM succinate, were found to express detectable amount of EGFP in the concentration range of 0.5 nM to 10 mM (**Figure 4A**). Compared to the uninduced culture, a small but detectable increase of relative fluorescence at 513 nm was observed for the whole cell (1.4 fold) and the cell lysate (2.2 fold) of 2NBA-induced cultures of BR^prox^ mutant at 1.0 and 0.5 nM, respectively. Thus it is clear that the constructed bioreporter is sensitive enough to detect 2NBA at nanomolar concentrations (**Figure 4B**). The fluorescence signal increases with an increase in 2NBA concentration and becomes nearly saturated in the concentration range of 1–8 mM. However, at 10 mM 2NBA, a relatively lower level of EGFP expression was noticed (∼1/2 fold as compared to 1 mM 2NBA induction), and was completely lost at 12 mM (**Figures 4A,B**). The above observations were further substantiated from the growth experiments with BR^prox^ mutant strain, where only a small growth (OD_600_ = 0.3) was revealed in presence of 12 mM of 2NBA compared to that of lower concentrations (1, 8, and 10 mM) exhibiting relatively higher growth (OD_600_ ∼1.0) upon overnight incubation in MSM supplemented with 7 mM of succinate. Similarly, for the wild type ST-14 strain, only a feeble growth was observed in presence of 12 mM 2NBA (data not shown). It is believed that at higher concentrations (≥ 12 mM), 2NBA exerts cytotoxic effect on the cells and therefore the BR^prox^ mutant cells prefer stress management over 2NBA-inducible *onb* gene expression. This proposition was reinforced from the results of qPCR analysis which revealed drastic reduction in the expression of *egfp* mRNA transcripts (a drop by 96% in comparison to that in 1 mM 2NBA induction) in BR^prox^ cells, induced in presence of 12 mM of 2NBA (Supplementary Figure S4). In addition, enzymatic assay of 3-hydroxyanthranilate dioxygenase (OnbC) activity in cell-free extracts of 2NBA-induced BR^dist^ cells (with intact *onbC* but disrupted *onbIGDB* gene segments) showing a decrease in OnbC activity at 10 mM compared to 8 mM and a complete loss of activity at 12 mM of 2NBA, confirming inhibition of *onb* gene induction at this concentration of 2NBA (Supplementary Figure S5).

### Confocal Imaging and Flow Cytometric Analysis

**FIGURE 5:**
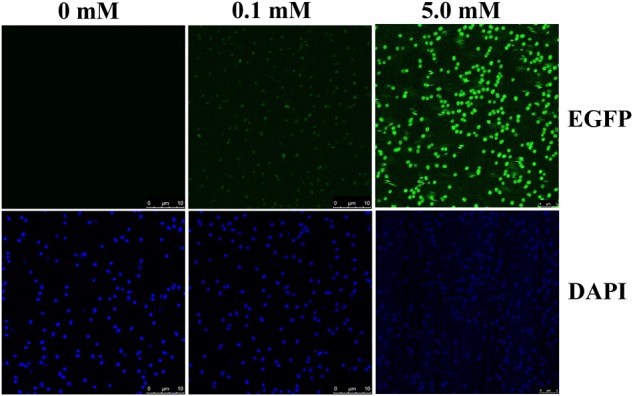
Confocal microscopy. Confocal microscopic images of BR^prox^ cells, induced with 0.1 mM and 5.0 mM of 2NBA. For visualization of internal EGFP (upper panel), cells were illuminated with 488 nm laser while cells stained with DAPI (lower panel) were analyzed using 356 nm laser. Cells without 2NBA induction were used as parallel controls.

**FIGURE 6:**
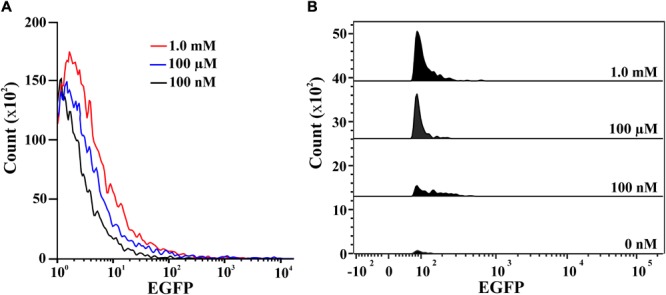
Flow cytometric analysis. **(A)** Overlay representation of the whole population of BR^prox^ cells induced with 2NBA at concentrations: 100 nM (black), 100 μM (blue) and 1.0 mM (red). **(B)** Stacked view of the EGFP expressing P2 population of BR^prox^ strain induced with 0 nM, 100 nM, 100 μM and 1.0 mM of 2NBA vis-à-vis respective relative EGFP intensity.

Confocal images of 2NBA-induced BR^prox^strain again confirmed the 2NBA concentration-dependent variation in EGFP expression by the reporter cells. The cells showed a significantly strong fluorescence (relative intensity 96 ± 1.7) when exposed to 5 mM of 2NBA, compared to its 50-fold lower concentration, where a very weak signal (relative intensity 27 ± 2.3) was detected (**Figure 5**). Thus the constructed bioreporter was able to produce distinguishable concentration-dependent signals. Additionally, flow cytometric analysis clearly depicted a rightward shift of the overall populations indicating, enhanced EGFP expression of the bioreporter cells, induced with the increasing concentration of 2NBA (**Figure 6A**). This observation was further strengthened by analyzing the EGFP expressing P2 populations of the same set of induced cells which exhibited successive increase in cell count with higher reporter signal (**Figure 6B**).

## Discussion

Two 2NBA-sensing bioreporters, mutant strains of wild type 2NBA biodegrader, *Cupriavidus* sp. ST-14 were constructed, which could successfully detect 2NBA based on the synthesis of the reporter protein EGFP. Generally, whole cell biosensors, used for the detection of a large variety of organic pollutants, are constructed in a bacterial system possessing a reporter plasmid harboring a well-characterized pollutant-responsive promoter and a regulator sequence, present upstream to a reporter gene (Stiner and Halverson, 2002; Tecon et al., 2009; Haque et al., 2013; Sun et al., 2017). Again, in most cases, *E. coli* acts as the reporting host (Willardson et al., 1998; Chang et al., 2004; Tecon and Van der Meer, 2008; Robbens et al., 2010; Behzadian et al., 2011) or the reporter plasmids are maintained in heterologous pollutant-degrading environmental biodegrader strains (Heitzer et al., 1992; Applegate et al., 1997; Layton et al., 1998; Stiner and Halverson, 2002). Nevertheless, plasmid-based bioreporters are advantageous because of their capability to produce a high level of reporter signal due to high copy number of the reporter gene present in multi-copy reporter plasmids (Hong and Park, 2014) and consequently, showing high sensitivity toward detecting low level of pollutants (van der Meer et al., 2004; Hong and Park, 2014). In spite of the above advantages, plasmid-based bioreporters suffer from usual problems associated with plasmid curing, inducer translocation and/or non-recognition of the exotic promoter sequence by sigma factors of the reporter hosts (van der Meer et al., 2004; Tecon and Van der Meer, 2008; Hong and Park, 2014; Gaida et al., 2015). To alleviate these problems, focus has shifted to the development of chromosome-based bioreporters where a reporter gene is incorporated into the host chromosome, placed under the control of an outsourced inducible promoter-operator sequence, pre-characterized for its strong inducer-responsive nature and regulated through host-intrinsic trans-acting regulator proteins (Hay et al., 2000; Hong and Park, 2014; Yagur-Kroll et al., 2015). Nevertheless, such bioreporter models, whether plasmid-based or chromosome-based, need appropriate sequence information of inducer-sensing promoter and the associated regulator. Nonetheless, with the advent of cloning and sequencing technology including whole genome sequencing facilities, it has become far easier to characterize structural genes of catabolic operons of bacterial origin but as such fail to obtain specific information on the identity and location of promoter-operator sequence and that of the role of regulator of the degradative operon. In such circumstances, it has been hypothesized that mere information of structural genes of a particular pollutant-inducible defined catabolic operon is enough to develop a bioreporter strain that can sense and quantify the target pollutant.

Capitalizing on the above promises, 2-nitrobenzoate-sensing whole cell bioreporter strains were constructed based on the molecular information of structural genes of the 2NBA-inducible *onb* catabolic gene cluster. At the same time, the construction of bioreporter was based on the assumption that the promoter-operator sequence should reside somewhere upstream to the first structural gene *onbF* (**Figure 1**), without prior knowledge on the identity and location of the promoter-operator sequence and the role of encoded regulator (Basu et al., 2016). A similar concept had been employed to construct a chromosome-based bioreporter to understand the mechanisms regulating the acetophenone-metabolic genes of an inducible catabolic operon in *Aromatoleum aromaticum* strain EbN1-SR7, although the constructed bioreporter exhibited broad substrate specificities toward acetophenone, propiophenone, ethylbenzene, 1-phenylethanol and all the monofluoroacetophenone isomers with a comparable sensitivity (Muhr et al., 2016). When subcultured in the absence of antibiotic stress, each day for a month, both the bioreporter strains BR^prox^ and BR^dist^ could retain the EGFP and Kan^R^ genes (data not shown) demonstrating a clear advantage over plasmid-based reporters that are susceptible to frequent loss of reporter gene due to plasmid curing under similar conditions. Again, these 2NBA-sensing bioreporters, constructed using the wild type ST-14 as the host strain, circumvent the issues associated with analyte transport and compatible sigma factor prevalent in plasmid-based bioreporter strains in *E. coli* or other heterologous hosts.

Unlike plasmid-based reporter systems, chromosome-based reporters often encounter issues with relatively poor sensitivity, with the detection limit in the micromolar to millimolar range due to the presence of a single copy reporter gene compared to usual high copy number for reporter plasmids. However, the reporter strain BR^prox^ was able to detect 2NBA with a lower detection limit of 0.5 nM for CFE and 1.0 nM for whole cell (**Figures 2C,4A**), exhibiting a better sensitivity compared to other reported chromosome-based bioreporters (Heitzer et al., 1992; Hay et al., 2000; van der Meer et al., 2004; Muhr et al., 2016). The higher sensitivity of BR^prox^ strain may be attributed to a strategic disruption of the first functional gene of the pathway i.e., *onbA* encoding a nitroreductase (that transforms 2NBA to 2-hydroxylaminobenzoate), permitting the presence of a steady level of intracellular 2NBA. Consequently, an effective induction occurred throughout the period of induction resulting in a higher expression level of the reporter gene. In addition, the proximal presence of the reporter gene to the hypothesized promoter region in the BR^prox^ strain has a significant effect on the sensitivity than that of the distally located reporter gene in BR^dist^ (**Figures 2A,B,C**). This may be due to polar effects where promoter proximal genes are better transcribed than the distal ones (Wek et al., 1987; Ciampi and Roth, 1988; Lim et al., 2011).

In the present study, optimum expression of reporter (EGFP) signals in BR^prox^ strain was obtained for stationary phase culture (**Figures 3D,E**). Unlike other reports using carbon-containing induction media (Applegate et al., 1997; Stiner and Halverson, 2002; Hong and Park, 2014; Muhr et al., 2016), EGFP signal in BR^prox^ cells could be detected both in the presence and absence of carbon source (**Figure 3B**) in the induction medium (Heitzer et al., 1992), establishing cost effectiveness on the applicability of the constructed bioreporter. It may be mentioned here that the presence of simpler carbon sources such as succinate in induction medium often delays start of desired induction until the easily degradable succinate gets depleted (Görke and Stülke, 2008). Hence, induction in carbon-free MSM enabled the BR^prox^cells to exhibit EGFP expression from the very 1st hour of induction, indicating a fast and efficient sensing response of the constructed reporter strain (**Figure 3C**). Again, for the selection of best growth phase cells for reporter signal expression, deployment of similar induction medium allowed to maintain the cells in their respective phases of growth at the time of induction.

Since the *onb* gene cluster is exclusively dedicated for the degradation of 2NBA, strict inducer specificity of the reporter strain toward 2NBA has also been reflected. Eventually, a failure to exhibit induction in the presence of *meta*- and *para*-isomers of 2NBA and its structurally related compounds (Supplementary Figure S3 and Supplementary Table S2) has made BR^prox^ advantageous over other bioreporters, which often showed cross-reactivity for closely related organic pollutants (Willardson et al., 1998; Stiner and Halverson, 2002; Haque et al., 2013; Muhr et al., 2016). Nonetheless, detectable EGFP expression in whole cell and cell lysate from 1.0 nM to 10 mM of 2NBA has enabled the bioreporter to respond over a wide concentration range while a nearly linear increase (*R*^2^ = 0.99776) in the reporter signal was observed up to 1.0 mM. Thus 2NBA concentration can be easily quantified in a contaminated sample based on reporter signal in the range of 1.0 nM to 1.0 mM (**Figure 4B**). However, EGFP expression was also detected at 0.5 nM of inducer concentration in the cell lysate of bioreporter strain (**Figure 4A**), keeping an application window open for 2NBA detection at subnanomolar levels. Apart from 2NBA quantification based on fluorescence spectral measurement and Western blot analysis, both confocal and flow cytometric data (**Figures 5, 6**) revealed easily distinguishable signals in the BR^prox^ cells treated with low and high concentrations of 2NBA.

Thus, in this study, approaches have been made to construct a highly specific and fairly sensitive bioreporter strain BR^prox^ by incorporation of the reporter gene *egfp* into the 2NBA degrading chromosomal *onb* gene cluster of the wild type strain *Cupriavidus* sp. ST-14 by disrupting the 2NBA transforming gene *onbA* without a prior knowledge on the identity and location of the promoter-operator sequence. To our knowledge, this is the first attempt to construct a bioreporter strain that can detect 2NBA over a wide range of concentration with high specificity. Moreover, the procedure and performance of the constructed reporter strain BR^prox^ have significant advantages over other plasmid-based or chromosome-based bioreporters reported so far in terms of ease of construction method, maintenance, cost, sensitivity as well as specificity. Therefore, the present study should serve as a template for possible construction of bioreporter strains for other environmental pollutants, which can not only pre-alert to environmental offenses and asses to prioritize the concentration-based contaminated environments but can also be used to monitor the degree of success of bioremediation.

## Author Contributions

SD and TD planned and designed the study, analyzed the data, and wrote the manuscript. SD and SB carried out the laboratory work. AS performed the fluorescence spectroscopic analysis. All authors contributed significantly to the interpretation of data, proofread the manuscript, and approved its submission.

## Conflict of Interest Statement

The authors declare that the research was conducted in the absence of any commercial or financial relationships that could be construed as a potential conflict of interest.
