# Supplementary material for: Development of a 2-Nitrobenzoate-Sensing Bioreporter Based on an Inducible Gene Cluster
Source: Front Microbiol. 2018 Feb 14;9:254. doi: 10.3389/fmicb.2018.00254 (PMC5817917; doi:10.3389/fmicb.2018.00254)
Supplement: Supplementary file 1 [file Data_Sheet_1.PDF]

Supplementary Material

Development of a 2-nitrobenzoate-sensing bioreporter based on an inducible gene cluster

Satamita Deb, Soumik Basu, Achintya Singha and Tapan K. Dutta[*]

*** Correspondence:** Tapan K. Dutta: tapan@jcbose.ac.in

1 Supplementary Tables and Figures

1.1 Supplementary Tables

Supplementary Table S1.

List of bacterial strains, plasmids, PCR amplified fragments and primers used in this study

**Bacterial strains**		
	***Cupriavidus* sp.**	
ST-14	Wild Type, 2NBA degrader, kanamycin sensitive (Kan^{s}); Length of the wild type *onb* genes are: *onbF* 450 bp (149 aa), *onbC* 540 bp (179 aa), *onbA* 651 bp (216 aa), *onbI* 1041 bp (346 aa), *onbG* 807 bp (268 aa), *onbD* 1023 bp (340 aa), *onbB* 519 bp (172 aa)	Basu et al., 2016
BR^{prox} (ST-14$^{\Delta onbFCA}$::EGFP)	Bioreporter, 2NBA^{-}EGFP^{+}Kan^{r}; site directed deletion mutant of ST-14 *onb* operon; *egfp* and *kanR* gene cassettes incorporated into the *onb* operon by complete deletion of *onbC* and partial deletion of *onbF* (89 aa from C-terminus) and *onbA* (138 aa from N-terminus)	This study
BR^{dist} (ST-14$^{\Delta onbIGDB}$::EGFP)	Bioreporter, 2NBA^{-} EGFP^{+}Kan^{r}; site directed deletion mutant of ST-14 *onb* operon; *egfp* and *kanR* gene cassettes incorporated into the *onb* operon by complete deletion of *onbG* and *onbD* and partial deletion of *onbI* (51 aa from C-terminus) and *onbB* (27 aa from N-terminus)	This study
	E. coli	
XL1 Blue	*recA1 endA1 gyrA96 thi-1 hsdR17 supE44 relA1 lac [F′ proABlacIq ZΔM15 Tn10 (Tet^{r})]*	
**Plasmids**		
pEGFP-C1	Mammalian expression vector containing *egfp* ; Kan^{r}; pUC_ori and f1_ori; CMV promoter	Clonetech, USA
pCM184	Allelic exchange vector; Amp^{r}; Kan^{r}; Cre/Lox	Addgene
pCM-prox	pCM184 harboring *onb* Up1 and *egfp* fragments in MCS1 and *onb* Down1 fragment in MCS2; Amp^{r}; Kan^{r}; Cre/Lox	This study
pCM-dist	pCM184 harboring *onb* Up2 and *egfp* fragments in MCS1 and *onb* Down2 fragment in MCS2; Amp^{r}; Kan^{r}; Cre/Lox	This study
**Amplified fragments**		
EGFP	Amplified from pEGFP-C1 vector,712 bp fragment with 5’ linker sequence containing stop codons in three reading frames and ribosome binding site	This study

Up1	Amplified from wild type ST-14 genomic DNA, 480 bp fragment located in *onb* operon from 1349 bp of *onbX2* to 179 bp of *onbF*	This study
Down1	Amplified from wild type ST-14 genomic DNA, 480 bp fragment located in *onb* operon from 415 bp of *onbA* to 218 bp of *onbR1*	This study
Up2	Amplified from wild type ST-14 genomic DNA, 540 bp fragment located in *onb* operon from 347-886 bp of *onbI*	This study
Down2	Amplified from wild type ST-14 genomic DNA, 540 bp fragment located in *onb* operon from 83 bp of *onbB* to 76 bp of *onbX3*	This study

Primers

Cloning Primers

Primer Name	Sequence (5' to 3') [a]	Cut Site
EGFP forward	CCG**GAATTC***TAATTAATTAAGAGCTACCGGTCGCCACC*AT GGTGAG	*EcoRI*
EGFP reverse	GGAATTC**CATATG**TTACTTGTACAGCTCGTCCATGCCG AGAGTG	*NdeI*
Up1 forward	GGA**AGATCT**GGCCTATGGGCCGATGTC	*BglII*
Up1 reverse	CCG**GAATTC**CGGATATCGACGCGCGAC	*EcoRI*
Down1 forward	TCC**CCGCGG**TGGGAATGCCGGCTGTTC	*SacII*
Down1 reverse	CG**ACGCGT** GGCACCACCCCGCGATTG	*MluI*
Up2 forward	GGA**AGATCT**CCGAGGCCGATATCTCGC	*BglII*
Up2 reverse	CCG**GAATTC**GGCGCAGGAAGCTCGAGT	*EcoRI*
Down2 forward	TCC**CCGCGG**ACGAGATCCGCTCGCGCC	*SacII*
Down2 reverse	CG**ACGCGT**CCGCGATGTCGAGCAGCT	*MluI*

RT Primers

Primer Name	Sequence (5' to 3')
EGFP_RT_F	CTTCTTCAAGTCCGCCATG
EGFP_RT_R	GGCGGATCTTGAAGTTCAC
onbC_RT_F	GGAATTTTTCTACCAGCTCAGG
onbC_RT_R	CTGTCGCAATACCATTCAAAGC
onbD-B_RT_F	CAGGTTCTTCAACTTGCCTG
onbD-B_RT_R	GGCACAGCTCCTGGTAAG
16S_RT_F	GGACAATGGGGGCAACC
16S_RT_R	ATTTCACGCCTGTCTTATCAAAC

[a] Restriction recognition sequences are in boldface type, and enhancer sequences are underlined (single solid line). EGFP 5' linker sequence is in italics underlined with single dashed line.

Supplementary Table S2.

List of compounds used for induction of BR^{prox} bioreporter strain

Compounds[a]	Induction		
	protein level[b]		mRNA level[c]
	RUD	RFU^{CFE}	RUD
2-Nitrobenzoate	40.81 ± 5.29	$32.2 \pm 0.85 \times 10^3$	15.26 ± 0.72
3-Nitrobenzoate	ND	$3.9 \pm 0.93 \times 10^3$	ND
4-Nitrobenzoate	ND	$4.1 \pm 1.1 \times 10^3$	ND
5-Hydroxy-2-nitrobenzoate	ND	$3.6 \pm 0.95 \times 10^3$	ND
2,4-Dinitrobenzoate	ND	$4.3 \pm 0.95 \times 10^3$	ND
2-Hydroxy-3,5-dinitrobenzoate	ND	$4.0 \pm 1.05 \times 10^3$	ND
Benzoate	ND	$4.2 \pm 0.85 \times 10^3$	ND
Gentisate	ND	$4.3 \pm 0.99 \times 10^3$	ND
Salicylate	ND	$3.9 \pm 1.12 \times 10^3$	ND
2-Chlorobenzoate	ND	$4.2 \pm 1.21 \times 10^3$	ND
2-Iodobenzoate	ND	$4.1 \pm 1.17 \times 10^3$	ND
2,3-Dihydroxybenzoate	ND	$3.7 \pm 0.98 \times 10^3$	ND
2-Hydroxy-5-methoxybenzoate	ND	$4.4 \pm 1.2 \times 10^3$	ND
2-Aminophenol	ND	$3.5 \pm 1.14 \times 10^3$	ND
2-Nitrophenol	ND	$3.5 \pm 0.97 \times 10^3$	ND
2-Nitrotoluene	ND	$3.6 \pm 1.29 \times 10^3$	ND
2,3-Dinitrotoluene	ND	$4.3 \pm 1.05 \times 10^3$	ND
2,4-Dinitrotoluene	ND	$3.9 \pm 1.09 \times 10^3$	ND
2-Nitroaniline	ND	$3.1 \pm 1.11 \times 10^3$	ND
2-Nitrobenzyl alcohol	ND	$3.5 \pm 1.15 \times 10^3$	ND
2-Nitrobenzaldehyde	ND	$4.1 \pm 0.91 \times 10^3$	ND
Phthalate	ND	$3.3 \pm 0.94 \times 10^3$	ND
2-Nitrobenzoyl chloride	ND	$4.4 \pm 0.99 \times 10^3$	ND
1-Chloro-2-nitrobenzene	ND	$4.3 \pm 1.05 \times 10^3$	ND
1-Fluoro-2-nitrobenzene	ND	$3.8 \pm 0.99 \times 10^3$	ND
2-Nitrophenyl acetate	ND	$4.4 \pm 1.25 \times 10^3$	ND
Methyl-2nitrobenzoate	ND	$4.3 \pm 1.19 \times 10^3$	ND
Succinate (Control)	ND	$3.5 \pm 1.6 \times 10^3$	ND

[a] All compounds were tested at 1 mM concentration (dissolved in methanol) for overnight induction; [b] Induction at protein level was analyzed by immunoblotting presented as RUD [Relative Unit of Densitometry ± SE (Standard Error) values] using 50 µg protein and by fluorescence analysis of cell-free extract (CFE) presented as RFU [Relative Fluorescent Unit ± SE (Standard Error) values] using 1 mg/ml of protein; [c] Induction at mRNA level was observed by RT PCR analysis keeping 16S rRNA as the endogenous control. ND, not detected (comparable to background RUD).

1.2 Supplementary Figures

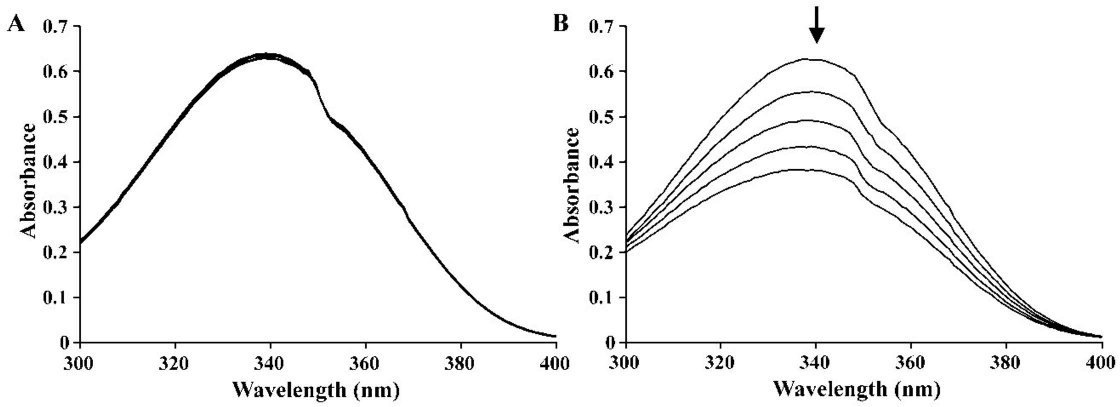

Supplementary Figure S1. Nitroreductase (OnbA) activity in bioreporter strains. The nitroreductase enzyme (translated from *onbA*) requires NADPH as co-factor for reduction of 2NBA to 2-hydroxylaminobenzoate with simultaneous oxidation of NADPH. OnbA activity in the cell-free extracts (CFE) of the 2NBA-induced BR^{prox} **(A)** and BR^{dist} **(B)** strains was determined spectrophotometrically by measuring NADPH oxidation at 340 nm. The sample and reference cuvettes contained 2NBA (0.04 mM each) and 50 µg of crude protein (CFE). The sample cuvette also contained 0.08 mM NADPH and absorbance was recorded at every one minute interval after the addition of crude protein. Vertical arrow indicates decreasing absorbance around 340 nm.

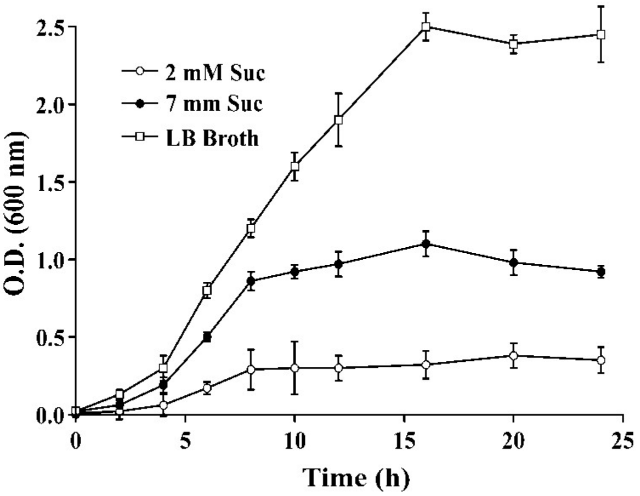

Supplementary Figure S2. Growth of BR^{prox} bioreporter strain. The BR^{prox} strain was grown in LB broth or MSM supplemented with either 2 mM or 7 mM succinate as carbon source over a period of 24 h. Growth (OD_{600}) was recorded at every 2 h interval. All growth medium contained

kanamycin (50 µg ml^{-1}) as selection marker. Vertical bars represent means ± standard deviations from triplicate biological measurements

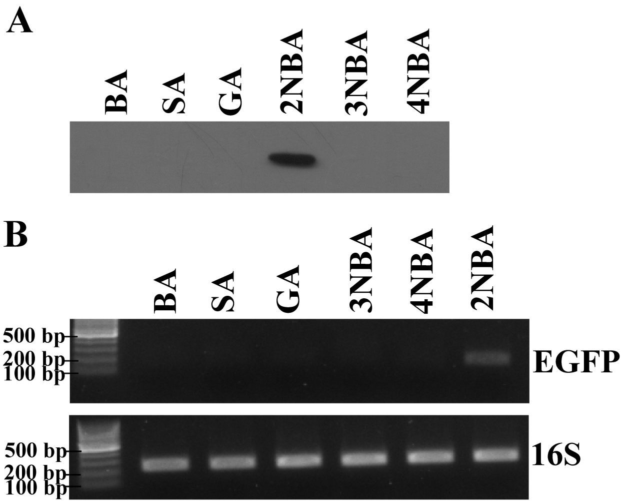

Supplementary Figure S3. Bioreporter specificity. (A) EGFP expression profiles of the bioreporter strain (BR^{prox}) induced with 2NBA, its structural isomers (3NBA and 4NBA) and other structurally related compounds (in MSM + 1 mM succinate), analyzed by immunoblotting using 50 µg protein from the respective cell lysates. **(B)** RT PCR analyses to detect mRNA transcript level of *egfp* gene in the same set of induced cells as described above, keeping *16S rRNA* transcript as the endogenous control. BA, benzoate; SA, salicylate; GA, gentisate; 2NBA, 2-nitrobenzoate; 3NBA, 3-nitrobenzoate; 4NBA, 4-nitrobenzoate.

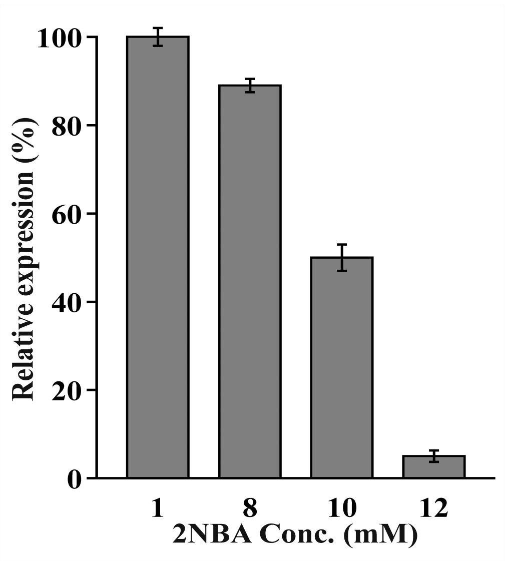

Supplementary Figure S4. Relative Expression of EGFP mRNA in 2NBA induced BR^{prox} cells. mRNA transcript levels of the reporter gene *egfp* were measured by qPCR in BR^{prox} cells induced with 2NBA at 1, 8, 10 and 12 mM concentrations. In the quantitative estimation, mRNA transcript level in cells induced with 1 mM of 2NBA has been accounted as 100%.

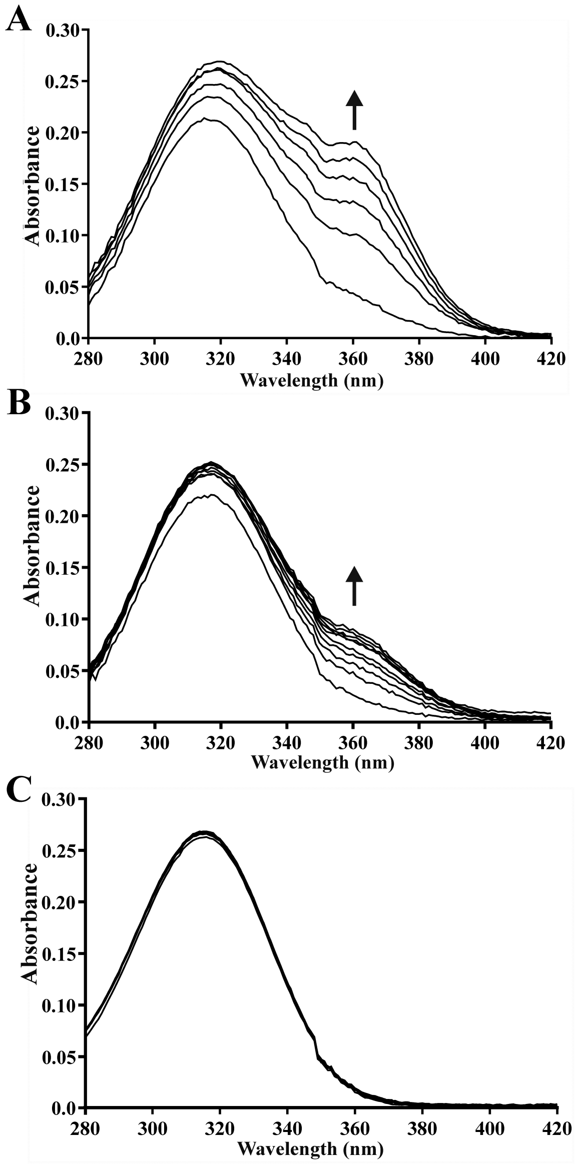

Supplementary Figure S5. 3-hydroxyanthranilate dioxygenase (OnbC) activity in BR^{dist} bioreporter strain. Spectrophotometric measurement of OnbC activity profiles in the cell-free extracts (CFE) of BR^{dist} cells induced in presence of 2NBA at concentrations of 8 mM **(A)**, 10 mM **(B)** and 12 mM **(C)**, based on the transformation of 3-hydroxyanthranilate (3HAA) to the ring cleaved product 2-amino-3-carboxymuconic-6-semialdyhyde resulting in an increase in the absorbance at 360 nm. The sample and reference cuvettes contained Fe^{2+} (0.01 mM), a dissociable cofactor present in the catalytic site of OnbC, and 100 µg of crude protein (CFE). Absorbance was recorded at every one minute interval after the addition of 3HAA (0.04 mM) to the sample cuvette. Vertical arrow indicates increasing absorbance around 360 nm.
